# Measurement of muscle blood flow and O_2_ uptake via near-infrared spectroscopy using a novel occlusion protocol

**DOI:** 10.1038/s41598-020-79741-w

**Published:** 2021-01-13

**Authors:** Joshua J. Dennis, Chad C. Wiggins, Joshua R. Smith, Jennifer M. J. Isautier, Bruce D. Johnson, Michael J. Joyner, Troy J. Cross

**Affiliations:** 1 Department of Anesthesiology and Perioperative Medicine, Mayo Clinic, Rochester, MN USA; 2 Department of Cardiovascular Diseases, Mayo Clinic, Rochester, MN USA; 3 Faculty of Medicine and Health, The University of Sydney, Sydney, NSW 2006 Australia

**Keywords:** Blood flow, Metabolism, Near-infrared spectroscopy

## Abstract

We describe here a novel protocol that sequentially combines venous followed by arterial occlusions to determine muscle blood flow and O_2_ uptake from a *single* measurement point using near-infrared spectroscopy (NIRS) during handgrip exercise. NIRS data were obtained from the flexor digitorum superficialis (FDS) muscle on the dominant arm of 15 young, healthy adults (3 women; 26 ± 7 years; 78.6 ± 9.1 kg). Participants completed a series of 15-s static handgrip contractions at 20, 40 and 60% of maximal voluntary contraction (MVC) immediately followed by either a: (i) venous occlusion (VO); (ii); arterial occlusion (AO); or venous then arterial occlusion (COMBO). Each condition was repeated 3 times for each exercise-intensity. The concordance correlation coefficient (CCC) and robust linear mixed effects modeling were used to determine measurement agreement between vascular occlusion conditions. FDS muscle blood flow (${\dot{\text{Q}}}_{{{\text{FDS}}}}$) and conductance (${\text{C}}_{{{\text{FDS}}}}$) demonstrated strong absolute agreement between VO and COMBO trials from rest up to 60%MVC, as evidenced by high values for CCC (> 0.82) and a linear relationship between conditions that closely approximated the line-of-identity (perfect agreement). Conversely, although FDS muscle O_2_ uptake (${{\dot {\text{V}}}}{{\text{O}}_{2{\text{FDS}}}}$) displayed “substantial” to “near perfect” agreement between methods across exercise intensities (i.e., CCC > 0.80), there was a tendency for COMBO trials to underestimate ${{\dot {\text{V}}}}{{\text{O}}_{2{\text{FDS}}}}$ by up to 7%. These findings indicate that the COMBO method provides valid estimates of ${\dot {\text{Q}}}_{{\text{FDS}}}$ and, to a slightly lesser extent, ${{\dot {\text{V}}}}{{\text{O}}_{2{\text{FDS}}}}$ at rest and during static handgrip exercise up to 60%MVC. Practical implications and suggested improvements of the method are discussed.

## Introduction

Near infrared spectroscopy (NIRS) is a noninvasive technology that can be used to assess the concentrations of oxy/deoxyhemoglobin ([HbO_2_] and [HHb]), and total hemoglobin ([THb]) of regional tissues, including the superficial skeletal muscles during exercise. Not only does this technology allow for in vivo assessment of tissue O_2_ status, one may obtain estimates of tissue blood flow and O_2_ uptake when NIRS is used in combination with venous and arterial occlusions, respectively^1–9^. Specifically, muscle blood flow is obtained from the ‘upslope’ in [THb] observed at the onset of venous occlusion (VO), whereas muscle O_2_ uptake is determined from the ‘downslope’ in [HbO_2_] during arterial occlusion (AO). The principal drawback of these methods is that they are not performed simultaneously; i.e., venous and arterial occlusions are performed following separate exercise bouts. In turn, this caveat necessarily lengthens the duration of any experimental design where an investigator seeks to quantify *both* muscle blood flow and O_2_ uptake under a given condition, or set of conditions (e.g., range of exercise intensities). To circumvent this limitation, we have developed an expedited protocol that sequentially combines both methods into a single measurement period. Specifically, our novel combined protocol (COMBO) consists of a brief period of venous occlusion, immediately followed by transient arterial occlusion such that both vascular perturbations are completed within < 15 s after exercise.

The aim of this report was to assess the measurement agreement between muscle blood flows, and O_2_ uptake values obtained via the VO and AO methods, separately, and those values obtained from our novel combination (COMBO) protocol. NIRS-derived absolute blood flows and O_2_ uptake of the flexor digitorum superficialis (FDS) muscle were assessed at rest, and during isometric handgrip exercise at 20, 40, and 60% of maximal voluntary contraction (MVC), ensuring that measurement agreement between methods was evaluated over a wide physiological range of values.

## Methods

### Ethical approval

Study procedures were approved by the Mayo Clinic Institutional Review Board (IRB# 19-000060) and conformed to the principles outlined in the *Declaration of Helsinki*. Participants provided written informed consent prior to beginning the study.

### Participants

Fifteen young, healthy adults (12 men and 3 women; 26 ± 7 years; 78.6 ± 9.1 kg; 176 ± 9 cm) volunteered to participate in this study and provided written informed consent. All participants were non-smokers and underwent a pre-participatory health screening to ensure they had no current, or history of, cardiac, pulmonary, and/or metabolic diseases. Participants were asked to refrain from caffeine intake and strenuous physical activity involving the forearms ~ 24 h prior to visiting the laboratory.

### Experimental design

To assess the measurement validity of our novel occlusion protocol across a physiological range of values, participants completed a series of incremental handgrip exercise protocols wherein muscle blood flow and O_2_ uptake was obtained by either the venous occlusion (VO), arterial occlusion (AO) or combined occlusion (COMBO) protocols. Participants were seated in a semi-recumbent position. The incremental handgrip protocol consisted of 15-s isometric contractions at forces equal to 20, 40 and 60% of participants’ maximal voluntary contraction (MVC). Each contraction was separated by 90 s of passive recovery. Immediately following the cessation of each contraction, either the venous, arterial or combined occlusions were applied to measure forearm muscle hemodynamics via NIRS (see below). Importantly, the specific occlusion condition (i.e., VO, AO, and COMBO) was constant for the duration of each incremental handgrip protocol (i.e., 3 × 15 s contractions at 20, 40 and 60%MVC). At the end of a complete incremental handgrip protocol, participants were given an extended period of passive recovery before performing the next protocol. Participants performed another MVC toward the end of this extended recovery. The length of recovery was 5 min or until NIRS variables and MVC returned to baseline values—which ever occurred first.

**Figure 1:**
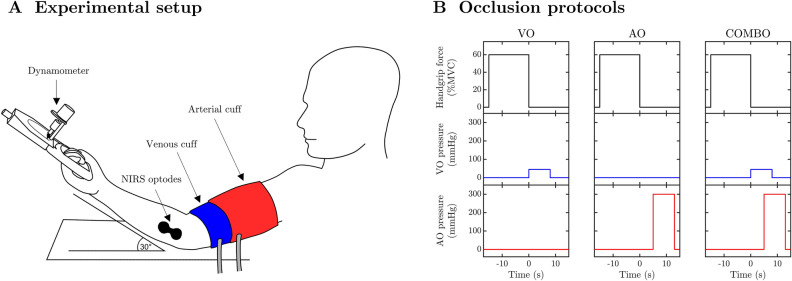
Illustration of the experimental design (**A**) and the venous, arterial and combined occlusion protocols (**B**). NIRS: near-infrared spectroscopy; VO: venous occlusion; AO: arterial occlusion; COMBO: combined venous and arterial occlusion; MVC: maximal voluntary contraction. The illustration in Panel A was created using InkScape (v0.92, Inkscape Project, https://inkscape.org).

Each occlusion condition was repeated 3 times in randomized order (i.e., a total of 9 incremental handgrip protocols and 27 static contractions). Handgrip exercise was performed using an isometric dynamometer (Smedley Hand Dynamometer, Stoelting Co., Wood Dale, IL) that was modified to provide an analog output proportional to handgrip force. This analog output was normalized to the participant’s MVC, and was used to provide visual feedback during contractions. An overview of the experimental setup is illustrated in Fig. 1A. Before commencement of the first handgrip protocol, the VO, AO and COMBO conditions were applied while the participant was seated at rest. Resting trials were repeated 5 times in random order. The entire experimental design was completed within 2.5 h during a single visit to the laboratory. Throughout the entire study, the participants’ dominant forearm was held in position at heart level with an upward angle of ~ 30° by use of a wedge-shaped arm rest.

### Blood pressure and heart rate

Beat-by-beat arterial blood pressure was measured using a pneumatic cuff placed around the middle phalanx of the non-dominant hand and was connected to a photoplethysmograph (Nexfin; BMEYE, Amsterdam, Netherlands). The non-dominant hand was placed at the side of the participant in a comfortable position, which was often below the level of the heart. The resulting hydrostatic pressure artefact was negated by a height sensor attached to the hand bearing the finger cuff. The finger pulse waveform was calibrated to brachial artery pressure at rest before the experimental protocol began. The mean arterial pressure (MAP) was taken as the arithmetic mean of finger pressure across the cardiac cycle. Cardiac rhythm and heart rate were also recorded using an external 3-lead electrocardiogram module.

### Near-infrared spectroscopy

NIRS data were obtained from the flexor digitorum superficialis (FDS) on the dominant forearm, using a fixed source-detector separation of 35 mm (Fig. 1A)^10^. Optodes were affixed to the skin with double-sided adhesive and covered by an optically-dense material to minimize confounding effects of ambient light. A continuous-wave near-infrared spectrophotometer (NIRO 200NX, Hamamatsu Photonics, Tokyo, Japan) was used to detect the light remitted from 3 laser diode sources of wavelengths 735, 810, and 850 nm. At these wavelengths, it is possible to differentiate between oxygenated and deoxygenated forms of hemoglobin and myoglobin^11^. The changes in near-infrared light absorption at each wavelength were converted to absolute changes in tissue concentrations of HbO_2_ and HHb using a modified Beer–Lambert law. The modified Beer–Lambert law incorporated a differential path-length factor to account for photon scattering in biological tissue. A fixed differential path-length factor of 4.0 was used in this study^12^. Total hemoglobin concentration (i.e. [THb]) was computed as the sum of [HbO_2_] and [HHb], whereas the hemoglobin difference ([HbDiff]) was determined by subtracting [HHb] from [HbO_2_]. Analog signals representing [HbO_2_] and [HHb] were outputted from the NIRS system at 20 Hz (the fastest setting).

### Venous, arterial and combined occlusion protocols

To apply the various occlusions, participants were instrumented with two straight pneumatic cuffs placed on the upper arm of the dominant side (SC10; Hokanson Inc., Bellevue, WA, USA). The cuff responsible for applying venous occlusions was placed on the arm first, whereby much care was taken to position the cuff as close as possible to the crease of the elbow (Fig. 1A). This relatively distal placement of the venous occlusion cuff obviated the “milking/jump” artefact that is sometimes observed in [THb] at the onset of cuff inflations. The pneumatic cuff responsible for applying the arterial occlusion was placed more proximal on the upper arm to ensure that the majority of all conduit arteries feeding the forearm were completely obstructed during inflations (Fig. 1A). Each pneumatic cuff was connected to two, separate, rapid cuff-inflator systems (E20, Hokanson Inc.). The inflation pressures for the venous and arterial occlusion cuffs were set to 40–50 mmHg and 300 mmHg, respectively. It is worth noting that the inflation pressure for arterial occlusions (300 mmHg) was relatively higher than we have used before (i.e., 50 mmHg above resting systolic blood pressure)^10^. This higher arterial cuff pressure was chosen to help facilitate a more rapid emptying of the inner venous occlusion cuff during the COMBO trials (see below). The positions of each pneumatic cuff were secured in place using athletic tape.

**Figure 2:**
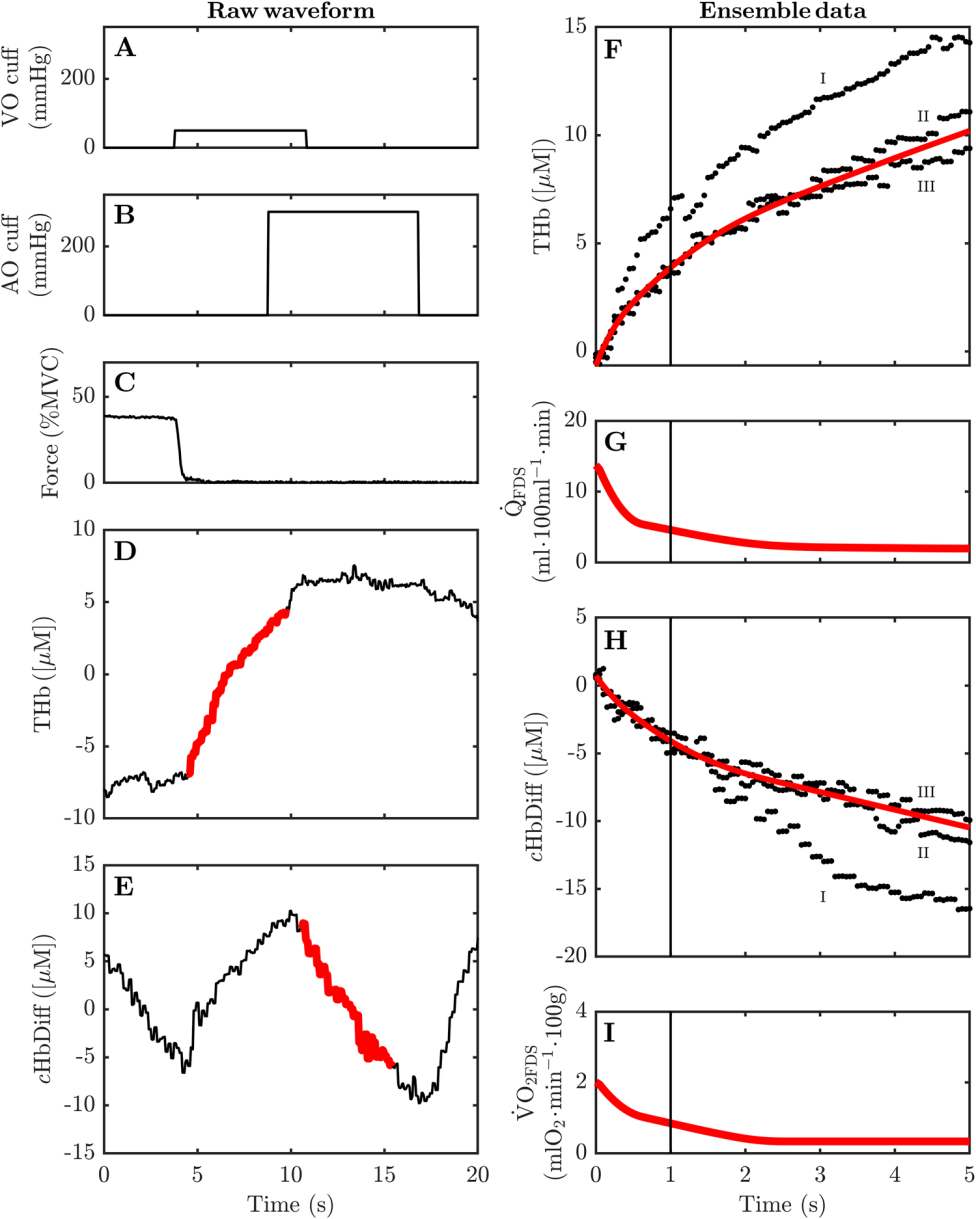
An example of the near-infrared waveform response to the combined venous and arterial occlusion following a 15-s isometric handgrip contraction. VO: venous occlusion; AO: arterial occlusion; MVC: maximal voluntary contraction; THb: total hemoglobin; *c*HbDiff: corrected hemoglobin difference; FDS: flexor digitorum superficialis; ${\dot {\text{Q}}}$: muscle blood flow; ${\dot {\text{V}} \text{O}}_{\text{2}}$: muscle O_2_ uptake. The solid lines in (**A**) and (**B**) denote the relative timing of venous and arterial occlusions during the combined protocol (COMBO). The solid lines in (**C**–**E**) represent the continuous waveform response to the COMBO occlusion protocol immediately following an isometric handgrip contraction at 40%MVC. The thickened red lines in (**D**) and (**E**) denote the periods of data used to compute ${\dot {\text{Q}}}$ and ${\dot {\text{V}} \text{O}}_{\text{2}}$, respectively, of the FDS muscle. The solid dots in (**F**) and (**H**) represent the ensemble of [THb] and [*c*HbDiff] waveforms obtained during COMBO occlusions following 40%MVC handgrip exercise in this particular individual. The numerals I, II and III in (**F**) and (**H**) illustrate from which of the 3 repeated trials waveform data were obtained. The solid red lines in (**F**) and (**H**) depict the best-fit nonlinear spline after the processes of iterative reweighting was complete. Note that the iterative reweighting approach has deemphasized the observations belonging to the aberrant waveform in each dataset. The solid red lines in (**G**) and (**I**) are the first-order derivatives obtained from the best-fit splines in (**F**) and (**H**). The vertical solid lines in (**F**–**I**) indicate the first 1-s of the COMBO occlusion over which the ${\dot {\text{Q}}}$ and ${\dot {\text{V}} \text{O}}_{\text{2}}$ waveforms were averaged.

The timing and length of vascular occlusions varied slightly depending on whether the venous or arterial occlusions were being applied separately as with the VO and AO conditions (Fig. 1B), or together as with the COMBO condition (Figs. 1B, 2A,B). Venous occlusions (VO) began immediately at the cessation of each static handgrip contraction and lasted for a duration of 8 s (Fig. 2C). Arterial occlusions (AO) began ~ 5 s after the cessation of handgrip exercise and lasted for 8 s. The delay of ~ 5 s at the start of the AO condition was required to keep its timing consistent with the AO cuff inflations during the COMBO condition (see next). The timing of events during the COMBO condition was identical to that described for the VO and AO conditions above, excepting that both were applied sequentially. In this manner, there was a 5-s window at the start of the COMBO condition where only VO was applied, thereafter the AO cuff was inflated, and for a further 5 s both cuffs were active. The overlap of cuff inflations during the COMBO condition served to reduce the time required for the AO cuff to achieve its final, suprasystolic pressure.

### Data collection

All signals were sampled continuously at 400 Hz (PowerLab 16/35, ADInstruments Inc., Colorado Springs, CO, USA) and stored on a personal computer for offline analysis. The timing of cuff inflations and deflations were controlled using the “Stimulator Panel” module available from within the software environment used for data acquisition (LabChart v8, ADInstruments Inc., Colorado Springs, CO, USA). All NIRS data were low-pass filtered using a zero-phase shift 3rd order Butterworth filter with a cut-off frequency of 40 Hz, after which data were down-sampled to 20 Hz for further analysis.

### Data analysis

#### Muscle blood flow and vascular conductance

Tissue blood flow of the FDS muscle (${\dot {\text{Q}}}_{{\text{FDS}}}$) was determined using the ‘upsloping’ response of [THb] waveform during periods of VO (Fig. 2D), as we and others have done^3,7,10,13^. Many investigators have calculated ${\dot {\text{Q}}}_{{\text{FDS}}}$ from the slope of a linear segment fit to the initial rise in [THb] after the onset of venous occlusion. Importantly, the width of this linear segment varies greatly in the literature (i.e., ~ 3–80 s)^1–3,5–8,13–15^. Importantly, by fitting a linear segment over any portion of the [THb] upslope, it is presumed that arterial inflow during venous occlusions remains constant over the same period. However, we have recently demonstrated that arterial inflow progressively declines throughout the duration of a venous occlusion, wherein the highest value of ${\dot {\text{Q}}}_{{\text{FDS}}}$ is typically observed during the first cardiac cycle after cuff inflation^10^. Unfortunately, in this study, we were unable to take a “beat-by-beat” approach to the analysis of ${\dot {\text{Q}}}_{{\text{FDS}}}$ due to the inadequate output sampling frequency of our NIRS device (20 Hz analog output). Nonetheless, we did not feel it was appropriate to fit a linear segment to any portion of the [THb] upslope during VO trials given the knowledge that ${\dot {\text{Q}}}_{{\text{FDS}}}$ rapidly declines after the onset of cuff inflation. Thus, we sought to circumvent the limitations incurred by a low output sampling frequency by time aligning several [THb] waveforms during VO trials, and fitting a nonlinear function to all available data. In brief, [THb] data were extracted from the first 5 s of each VO trial. These data were concatenated into a single array of *xy* pairs of time and [THb] values. A shape-constrained, piecewise cubic spline was fit to this pooled data of the participant^16^, and a continuous approximation of the average [THb] upslope during VO was obtained (Fig. 2F). The number of spline knots was set at 12 because preliminary results indicated that more knots unduly increased the complexity of the curve (risking overfitting), whereas a lesser number of knots was not sufficient to capture rapid changes in curvature present at the beginning of venous occlusions. The spline was “shape-constrained” to be a monotonically increasing, downwardly concaved function with a smooth 3rd order derivative. It became apparent early on during data analysis that although there was, on average, a degree of uniformity in the [THb] response to venous occlusions across the 3 repeated trials, we encountered several instances where the [THb] waveform would markedly deviate from the trend of the other two. This problem can be observed in the example provided in Fig. 2F, whereby one trial is quite clearly aberrant as compared with the other two (trial I). To ensure that the fitted spline was not unduly influenced by such outliers, we employed an iterative reweighting scheme to the curve fitting process. This method is summarized by the following steps:

1. Fit the pooled data with an initial shape-constrained piecewise cubic spline.
2. Compute observation weights using the residual scores (we used the bi-square weighting function in this study)^17^.
3. Refit the spline to the original data using the updated observation weights.
4. Repeat step 3 until the model coefficients converge or a maximum number of iterations have been reached.
5. Use the final set of observation weights to fit the spline to the original data.

Because the final spline could be expressed as a series of piecewise cubic functions, the first-order derivative of the [THb] spline could be solved analytically. The first-derivative of the [THb] spline ($[\text{T} \dot {\text{H}} \text{b}]$) was averaged over the first 1-s interval (Fig. 2G). We opted for using this mean over the first 1-s rather than reporting the instantaneous peak $[\text{T} \dot {\text{H}} \text{b}]$ at the beginning of the venous occlusion. We found that the 1-s average of $[\text{T} \dot {\text{H}} \text{b}]$ was more stable and less prone to instability of curve fitting at the boundaries of the data window (i.e., *t* = 0 s). The average $[\text{T} \dot {\text{H}} \text{b}]$ was converted to an absolute value of ${\dot {\text{Q}}}_{{\text{FDS}}}$ in units of ml min^−1^ 100 ml using a total hemoglobin count of 7.5 and 8.5 mmol L^−1^ for female and male participants, respectively^1^. The molecular mass of hemoglobin (64.458 g mol^−1^) and the ration between hemoglobin and O_2_ molecules (1:4) were accounted for in the calculation of ${\dot {\text{Q}}}_{{\text{FDS}}}$. The same approach was used to compute ${\dot {\text{Q}}}_{{\text{FDS}}}$ during the VO portion of the COMBO. To differentiate between these two quantities, we hereafter refer to the ${\dot {\text{Q}}}_{{\text{FDS}}}$ obtain during the VO and COMBO trials as ${\dot {\text{Q}}}_{{\text{FDS(VO)}}}$ and ${\dot {\text{Q}}}_{{\text{FDS(COMBO)}}}$, respectively. In an effort to account for variations in perfusion pressure between consecutive trials, the vascular conductance of the FDS muscle ($\text{C}_{{\text{FDS}}}$) was computed by taking the product of ${\dot {\text{Q}}}_{{\text{FDS}}}$ and the average MAP during venous occlusions. A similar naming convention was used to differentiate $\text{C}_{{\text{FDS}}}$ determined via the VO and COMBO trials (i.e., $\text{C}_{{\text{FDS(VO)}}}$ and $\text{C}_{{\text{FDS(COMBO)}}}$, respectively).

#### *Muscle O*_*2*_* uptake*

Tissue O_2_ uptake of the FDS muscle (${{\dot {\text{V}}}}{{\text{O}}_{2{\text{FDS}}}}$) was determined during periods of AO (Fig. 2E) in a similar manner to that described above for ${\dot {\text{Q}}}_{{\text{FDS}}}$. Before ${{\dot {\text{V}}}}{{\text{O}}_{2{\text{FDS}}}}$ was computed, it was first necessary to correct the [HbO_2_] and [HHb] waveforms for changes in tissue blood volume (i.e., [THb]) during the period of arterial occlusion^18^. These corrected waveforms were used to compute a blood-volume corrected hemoglobin difference ([*c*HbDiff]). The iterative reweighting scheme was applied the pooled [*c*HbDiff] waveforms taken from the initial 5-s period of the 3 repeated AO trials (Fig. 2H). Importantly, however, this spline was shape-constrained to be a monotonically decreasing, upwardly concaved function with a smooth 3rd order derivative. The first-order derivative of the final [*c*HbDiff] spline ($[c{{\text{H} \dot {\text{b}} \text{Diff}}}]$) was computed as described earlier for $[\text{T} \dot {\text{H}} \text{b}]$ (Fig. 2I). The average $[c \text{H} \dot {\text{b}} \text{Diff}]$ during the initial 1-s of arterial occlusion was converted to an absolute value of ${{\dot {\text{V}}}}{{\text{O}}_{2{\text{FDS}}}}$ (ml O_2_ 100 g^−1^ min) using the following expression:

1 $$ {\dot {\text{V}} \text{O}}_{{\text{2FDS}}} = \left\| {\left( {\left( {{{\left( {\frac{{\text{[}c{\text{H} \dot {\text{b}} \text{Diff}]}}}{2} \times 60} \right)} \mathord{\left/ {\vphantom {{\left( {\frac{{\text{[}c{\text{H} \dot {\text{b}} \text{Diff}]}}}{2} \times 60} \right)} {\left( {10 \times 1.04} \right)}}} \right. \kern-\nulldelimiterspace} {\left( {10 \times 1.04} \right)}}} \right) \times 4} \right)} \right\| \times {{22.4} \mathord{\left/ {\vphantom {{22.4} {1000}}} \right. \kern-\nulldelimiterspace} {1000}} $$

The muscle O_2_ uptake values obtained during the arterial occlusions of the AO and COMBO trials are denoted as ${\dot {\text{V}} \text{O}}_{{\text{2FDS(AO)}}}$ and ${\dot {\text{V}} \text{O}}_{{\text{2FDS(COMBO)}}}$, respectively.

### Statistical analyses

#### Goodness-of-fit statistics

The goodness-of-fit for the spline curves fit to the pooled [THb] and [*c*HbDiff] waveforms were evaluated by calculating the root-mean-squared error (RMSE), the adjusted coefficient of determination ($R_{{\text{adj}}}^{\text{2}}$) and the coefficient of variation of residual errors (CV) for each occlusion condition (i.e., VO, AO and COMBO) at each level of isometric handgrip exercise (i.e., rest and 20, 40 and 60%MVC).

#### Average responses

Two-way repeated measures analyses of variance were used to examine potential differences in MAP, HR, ${\dot {\text{Q}}}_{{\text{FDS}}}$ and $\text{C}_{{\text{FDS}}}$ between occlusion condition (i.e., VO *v* COMBO) at each intensity of isometric handgrip exercise (i.e., rest and 20, 40 and 60%MVC). A similar approach was taken to assess mean differences in ${{\dot {\text{V}}}}{{\text{O}}_{2{\text{FDS}}}}$ between occlusion conditions (i.e., AO *v* COMBO) at each of the 4 levels of isometric handgrip exercise-intensity.

#### Measurement agreement

The average error (bias), RMSE, typical error and concordance correlation coefficient (CCC) were all together used to assess the measurement agreement between VO and COMBO trials for ${\dot {\text{Q}}}_{{\text{FDS}}}$ and $\text{C}_{{\text{FDS}}}$, and between AO and COMBO trials for ${{\dot {\text{V}}}}{{\text{O}}_{2{\text{FDS}}}}$ at each exercise-intensity, separately. The average error was used to indicate systematic bias between two occlusion conditions, whereas the RMSE and typical error (TE) were taken as measures of the dispersion of error around the bias (i.e., precision). The CCC is a scaled index of the agreement between two methods^19^, ranging from − 1 (perfect disagreement), 0 (no agreement) to 1 (perfect agreement). In essence, the CCC is a measure of how well the relationship between two methods approximates the line-of-identity. The strength of “agreement” was qualitatively assessed by interpreting the CCC using a similar criteria that is usually applied to Pearson’s correlation coefficient (*r*) and Cohen’s kappa (κ): that is, slight agreement: 0.10 < CCC ≤ 0.20; fair agreement: 0.20 < CCC ≤ 0.40; moderate agreement: 0.40 < CCC ≤ 0.60; substantial agreement: 0.60 < CCC ≤ 0.80; near perfect agreement: 0.80 < CCC ≤ 0.99; perfect agreement: CCC > 0.99.

The measurement agreement between two occlusion methods was also assessed over the entire range of observed data using a robust linear mixed effects (LME) model. In brief, the linear mixed effects approach estimates the intercepts and slopes of a line explaining the relationship between two variables for each individual participant, while simultaneously estimating a single group-level intercept and slope. In this manner, one leverages the information afforded by repeated observations within an individual (i.e., 4 observations at different exercise intensities) to provide a more confident estimate of the linear relationship at the group-level. The robust LME extends this concept further by taking the iterative reweighting scheme described above for the [THb] and [*c*HbDiff] curve fits. In this study, we used the bi-square weighting function to fit robust LME models to ${\dot {\text{Q}}}_{{\text{FDS(VO)}}}$ *v* ${\dot {\text{Q}}}_{{\text{FDS(COMBO)}}}$, and $\text{C}_{{\text{FDS(VO)}}}$ and $\text{C}_{{\text{FDS(COMBO)}}}$, and ${\dot {\text{V}} \text{O}}_{{\text{2FDS(AO)}}}$ *v* ${\dot {\text{V}} \text{O}}_{{\text{2FDS(COMBO)}}}$ datasets, separately.

All data and statistical analyses were performed using MATLAB (The MathWorks Inc., Natick, MA, USA) and SPSS 26.0 (IBM, Armonk, NY, USA). Any pairwise inferences were adjusted for multiple comparisons using the Tukey-Sidak *post-hoc* adjustment. Statistical analyses were considered significant if *P* < 0.05.

## Results

### Goodness-of-fit statistics

The goodness-of-fit for the iteratively reweighted, piecewise cubic splines fit to [THb] and [*c*HbDiff] waveforms of each occlusion condition and exercise-intensity are reported in Table 1. No formal statistical comparisons were made on these data and, as such, are interpreted herein as strictly *qualitative* indicators of how well the curve fitting process described the data at hand. Overall, the goodness-of-fit of the [THb] spline curves was poorest at rest, and tended to improve at higher intensities of isometric handgrip force. Although RMSE appeared to increase with rising exercise-intensity, the increased variation of residual scores occupied a smaller fraction of the overall mean [THb] during occlusions, resulting in lower CV and higher $R_{{\text{adj}}}^{\text{2}}$ values at 20, 40, and 60%MVC compared with rest. Importantly, however, the RMSE, CV and $R_{{\text{adj}}}^{\text{2}}$ appeared similar between VO and COMBO trials. A similarly poor goodness-of-fit was observed for the [cHbDiff] splines at rest for both AO and COMBO trials (i.e., high CV and low $R_{{\text{adj}}}^{\text{2}}$). However, fitting performance tended to improve with increasing exercise-intensity. Intriguingly, the goodness-of-fit for [*c*HbDiff] splines appeared marginally worse for COMBO than for AO trials at each level of exercise-intensity.

**Table 1:** Goodness-of-fit statistics for the curve fitting procedures applied on the total haemoglobin ([THb]) and corrected haemoglobin difference ([*c*HbDiff]) waveforms during the venous, arterial and combined occlusions.

	VO				COMBO			
	Rest	20%MVC	40%MVC	60%MVC	Rest	20%MVC	40%MVC	60%MVC
**[THb]**								
RMSE_w_ (µM)	0.19 ± 0.03	0.38 ± 0.06	0.64 ± 0.11	0.65 ± 0.11	0.18 ± 0.03	0.49 ± 0.12	0.85 ± 0.15	0.64 ± 0.10
CV_w_ (%)	23.4 ± 3.4	11.8 ± 2.3	12.4 ± 3.8	5.7 ± 0.8	30 ± 4.6	11.2 ± 1.4	11.8 ± 16	6.6 ± 0.8
$R_{{\text{adj}}}^{\text{2}}$(%)	38.9 ± 19.8	80.2 ± 5.8	80.2 ± 8.7	97.4 ± 2.3	20.4 ± 38.8	80.5 ± 7.0	84.8 ± 8.2	93.8 ± 1.8

	AO				COMBO			
	Rest	20%MVC	40%MVC	60%MVC	Rest	20%MVC	40%MVC	60%MVC
**[cHbDiff]**								
RMSE_w_ (µM)	0.34 ± 0.07	0.56 ± 0.10	0.75 ± 0.16	0.79 ± 0.16	0.28 ± 0.07	0.71 ± 0.17	1.04 ± 0.22	1.04 ± 0.23
CV_w_ (%)	41.6 ± 11.3	12.3 ± 1.5	12.2 ± 2.2	10.6 ± 2.0	30.5 ± 5.7	19.5 ± 4.4	19.0 ± 3.9	15.0 ± 3.0
$R_{{\text{adj}}}^{\text{2}}$(%)	26.0 ± 15.8	80.5 ± 6.1	86.6 ± 3.9	87.5 ± 4.0	40.2 ± 12.4	67.6 ± 10.1	70.2 ± 8.7	74.6 ± 8.9

Values represent means ± SEM. VO, AO and COMBO: venous, arterial and combined occlusions, respectively. MVC: maximal voluntary contraction; RMSE_w_: weighted root mean squared error; CV_w_: weighted coefficient of variation; $R_{{\text{adj}}}^{\text{2}}$: Adjusted coefficient of determination.

### Average responses

**Figure 3:**
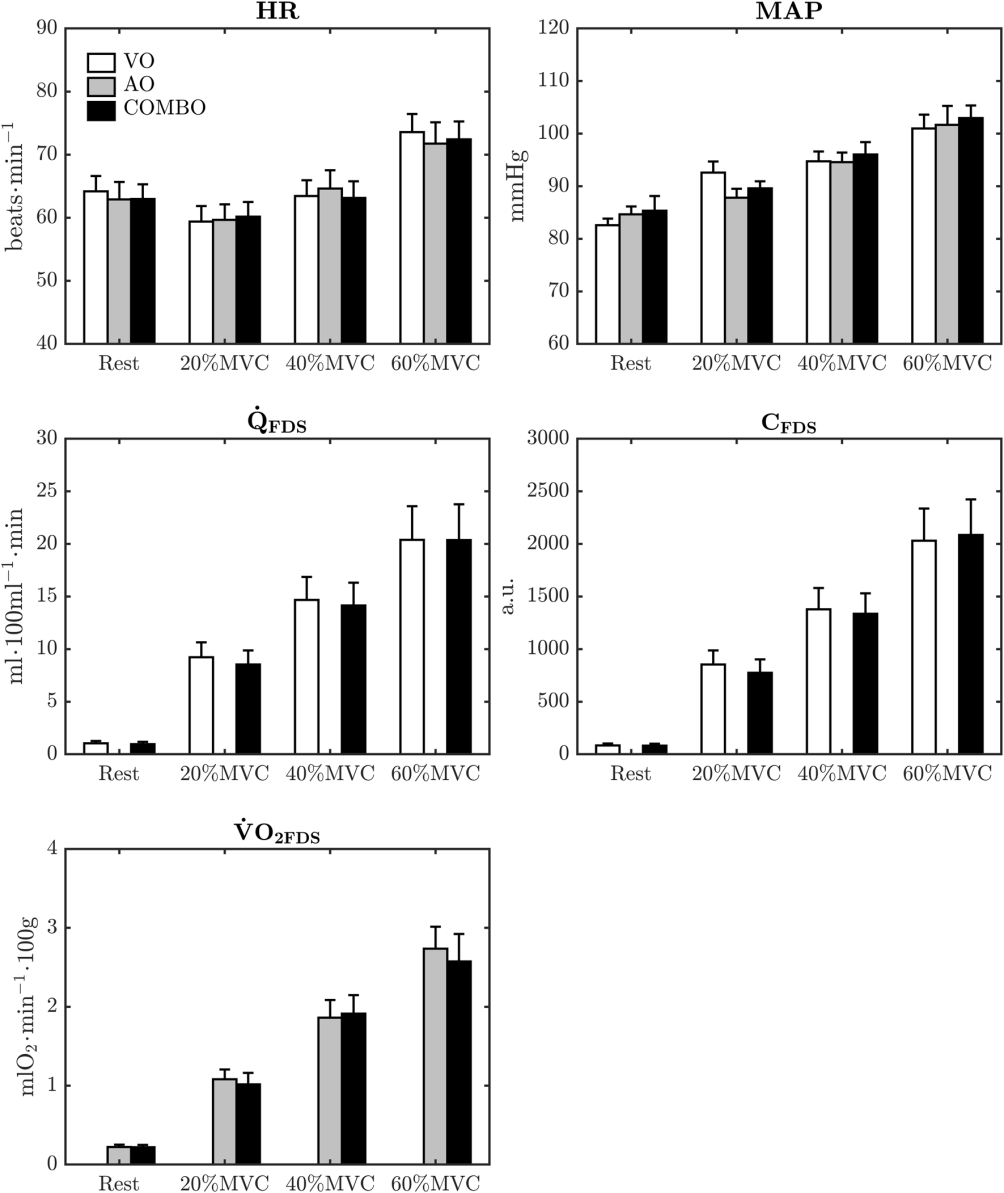
Average responses in hemodynamic and metabolic variables during incremental handgrip exercise. Bars represent means ± SEM. HR: heart rate; MAP: mean arterial pressure; FDS: flexor digitorum superficialis muscle; ${\dot {\text{Q}}}$: muscle blood flow; C: vascular conductance; ${\dot {\text{V}} \text{O}}_{\text{2}}$: muscle O_2_ uptake; MVC: maximal voluntary contraction. VO: venous occlusion; AO: arterial occlusion; COMBO: combined venous and arterial occlusion; a.u.; arbitrary units.

The group-average MVC at baseline was 499 ± 10 N and, as such, the prescribed isometric forces for the 20, 40 and 60%MVC contractions were 99 ± 4 N, 199 ± 8 N, and 299 ± 13 N, respectively. Participant’s MVC did not significantly change over the duration of the entire study. Figure 3 displays the average responses in cardiovascular variables and NIRS-derived hemodynamics across isometric handgrip exercise-intensity. A main-effect of exercise-intensity on average MAP, ${\dot {\text{Q}}}_{{\text{FDS}}}$, $\text{C}_{{\text{FDS}}}$ and ${{\dot {\text{V}}}}{{\text{O}}_{2{\text{FDS}}}}$ responses to isometric handgrip exercise was observed (*P* < 0.05). Here, MAP, ${\dot {\text{Q}}}_{{\text{FDS}}}$, $\text{C}_{{\text{FDS}}}$ and ${{\dot {\text{V}}}}{{\text{O}}_{2{\text{FDS}}}}$ demonstrated a consistent increase in magnitude from rest to 60%MVC (*P* < 0.05). The change in HR across exercise-intensity was less consistent, whereby its magnitude was only different from rest at 60%MVC (*P* < 0.05). Importantly, however, we observed no main effect for occlusion condition, nor any interaction between occlusion condition and exercise-intensity, for any of the above variables.

### Measurement agreement

The indices of absolute measurement agreement, bias, RMSE, TE and CCC for ${\dot {\text{Q}}}_{{\text{FDS}}}$, $\text{C}_{{\text{FDS}}}$ and ${{\dot {\text{V}}}}{{\text{O}}_{2{\text{FDS}}}}$ between the corresponding occlusion conditions, and across the handgrip exercise intensities are reported in Table 2. Overall, there was a negative systematic bias in the ${\dot {\text{Q}}}_{{\text{FDS}}}$ and $\text{C}_{{\text{FDS}}}$ measured by the COMBO compared with the VO trial, excepting that of $\text{C}_{{\text{FDS}}}$ at 60%MVC which was, in contrast, higher for the COMBO condition. There was a tendency for RMSE and TE of the differences in ${\dot {\text{Q}}}_{{\text{FDS}}}$ and $\text{C}_{{\text{FDS}}}$ between conditions to get larger with isometric handgrip exercise, indicating a slight reduction in precision. At all levels of exercise-intensity (rest, 20, 40 and 60%MVC), the CCC of the relationships between ${\dot {\text{Q}}}_{{\text{FDS(VO)}}}$ and ${\dot {\text{Q}}}_{{\text{FDS(COMBO)}}}$, and $\text{C}_{{\text{FDS(VO)}}}$ and $\text{C}_{{\text{FDS(COMBO)}}}$ demonstrated near-perfect agreement (i.e., 0.80 < CCC ≤ 0.99). The absolute measurement agreement in ${{\dot {\text{V}}}}{{\text{O}}_{2{\text{FDS}}}}$ between AO and COMBO trials ranged from substantial at rest and 20%MVC (i.e., 0.60 < CCC ≤ 0.80) to near-perfect agreement at 40 and 60%MVC (i.e., 0.80 < CCC ≤ 0.99).

**Table 2:** Measurement agreement for muscle blood flow and O_2_ uptake between the venous, arterial and combined occlusions.

	$\Delta {\dot{\text{Q}}}_{{{\text{FDS}}}}$(ml 100 ml^−1^ min)				ΔC_FDS_ (a.u.)				$\Delta {\dot{\text{V}}\text{O}}_{{2{\text{FDS}}}}$(ml min^−1^ 100 g)			
	Rest	20%MVC	40%MVC	60%MVC	Rest	20%MVC	40%MVC	60%MVC	Rest	20%MVC	40%MVC	60%MVC
Bias	– 0.07	– 0.70	– 0.53	– 0.01	– 0.9	– 91.8	– 49.1	72.8	– 0.01	–0.06	–0.05	–0.16
RMSE	0.36	1.50	4.03	2.40	40.0	160.0	420.3	271.8	0.06	0.35	0.29	0.61
TE	0.26	0.98	2.94	1.76	22.9	95.1	307.2	192.7	0.05	0.25	0.21	0.43
CCC	0.89	0.95	0.86	0.98	0.88	0.94	0.82	0.97	0.79	0.73	0.93	0.85

FDS: flexor digitorum superficialis muscle; $\Delta { \dot {\text{Q}}}$ and $\Delta  \text{C}$: differences in muscle blood flow and vascular conductance between venous (VO) and combined (COMBO) occlusion trials, respectively (i.e., COMBO–VO); $\Delta  { \dot {\text{V}} \text{O}}_{\text{2}}$: difference in muscle O_2_ uptake between arterial occlusion (AO) and COMBO trials (i.e., COMBO–AO); MVC: maximal voluntary contraction; RMSE: root mean squared error of the differences; TE: typical error; CCC: concordance correlation coefficient.

**Figure 4:**
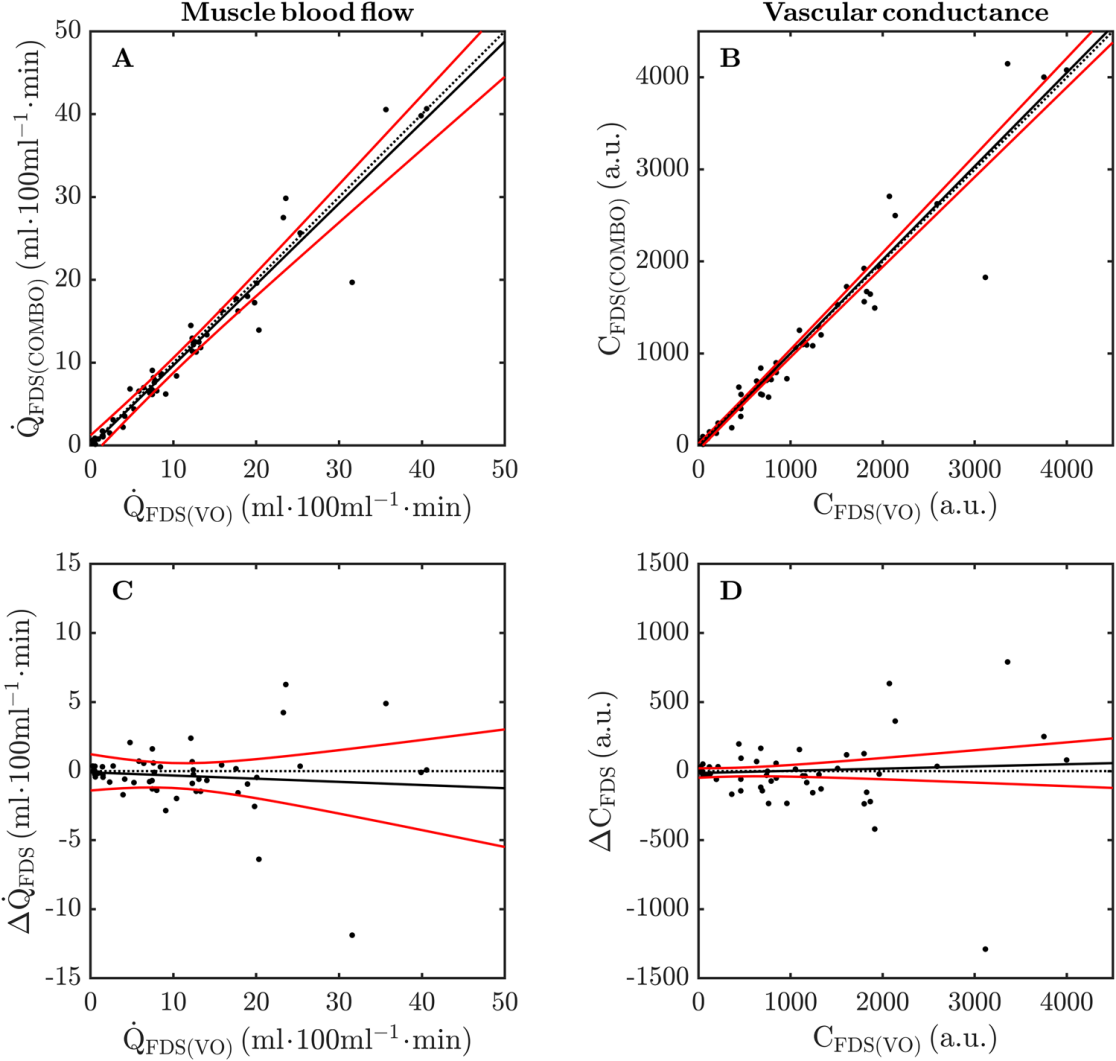
Measurement agreement between venous and combined occlusion trials for muscle blood flow (**A**,**C**) and vascular conductance (**B**,**D**) of the flexor digitorum muscle (FDS) during isometric handgrip exercise. Solid points represent individual data points from all participants at rest, and immediately following isometric handgrip exercise at 20, 40 and 60% of maximal voluntary contraction. The dotted lines denote the line-of-identity (i.e., perfect agreement). The solid lines represent the predicted group-level response obtained via robust linear mixed effects modeling. The solid red lines represent simultaneous 95% confidence intervals of the predicted group-level response. VO: venous occlusion; COMBO: combined venous and arterial occlusion; $\Delta { \dot {\text{Q}}}$ and $\Delta  \text{C}$: differences in muscle blood flow and vascular conductance between VO and COMBO trials, respectively (i.e., COMBO–VO).

The measurement agreement in ${\dot {\text{Q}}}_{{\text{FDS}}}$ and $\text{C}_{{\text{FDS}}}$ between VO and COMBO trials across the *entire range* of data is presented in Fig. 4A,B. In general, there appeared a very high level of absolute agreement in ${\dot {\text{Q}}}_{{\text{FDS}}}$ and $\text{C}_{{\text{FDS}}}$ as evidenced by the relatively narrow 95% simultaneous confidence intervals (CI_95%_) which encompassed the line-of-identity across the entire range of observed values. Figure 4C,D illustrate this point further whereby the simultaneous CI_95%_ of the robust LME model included zero over the whole range of data. Table 3 presents the parameter estimates of the robust LME model fit to the entire ${\dot {\text{Q}}}_{{\text{FDS(VO)}}}$ and ${\dot {\text{Q}}}_{{\text{FDS(COMBO)}}}$, and $\text{C}_{{\text{FDS(VO)}}}$ and $\text{C}_{{\text{FDS(COMBO)}}}$ relationships, separately. In both models, the slope estimates were different from zero (*P* < 0.05) whereas the intercept term was not. Accordingly, one may state from these results that ${\dot {\text{Q}}}_{{\text{FDS}}}$ determined via the COMBO condition marginally *underestimated* those values obtained using the VO condition by roughly ~ 1%. On the other hand, the $\text{C}_{{\text{FDS}}}$ obtained from the COMBO trials tended to *overestimate* those values derived from the AO condition by approximately 2%.

**Table 3:** Linear mixed effects model of the relationship between muscle blood flow via the venous (VO) and combined occlusion (COMBO) methods, and of the relationship between muscle O_2_ uptake obtained via the arterial (AO) and COMBO methods.

Variable	Comparison	Slope	Slope_var_	Intercept	Intercept_var_	RMSE	Adjusted R^2^
${\dot {\text{Q}}}_{{\text{FDS}}}$	COMBO v VO	0.99 ± 0.01*	0.000	– 0.25 ± 0.09	0.000	1.02	99.0
$\text{C}_{{\text{FDS}}}$	COMBO v VO	1.02 ± 0.01*	0.000	– 29.1 ± 17.7	16.8	111.5	98.9
${{\dot {\text{V}}}}{{\text{O}}_{2{\text{FDS}}}}$	COMBO v AO	0.93 ± 0.04*	0.021	0.01 ± 0.02	0.002	0.21	97.0

Values represent means ± SEM. FDS: flexor digitorum superficialis muscle; ${\dot {\text{Q}}}$ and C: muscle blood flow and vascular conductance, respectively;${\dot {\text{V}} \text{O}}_{\text{2}}$: muscle O_2_ uptake. Var: variance; RMSE: root mean squared error of the residual scores; *R*^2^: coefficient of determination.

*Significant parameter estimate, *P* < 0.05.

**Figure 5:**
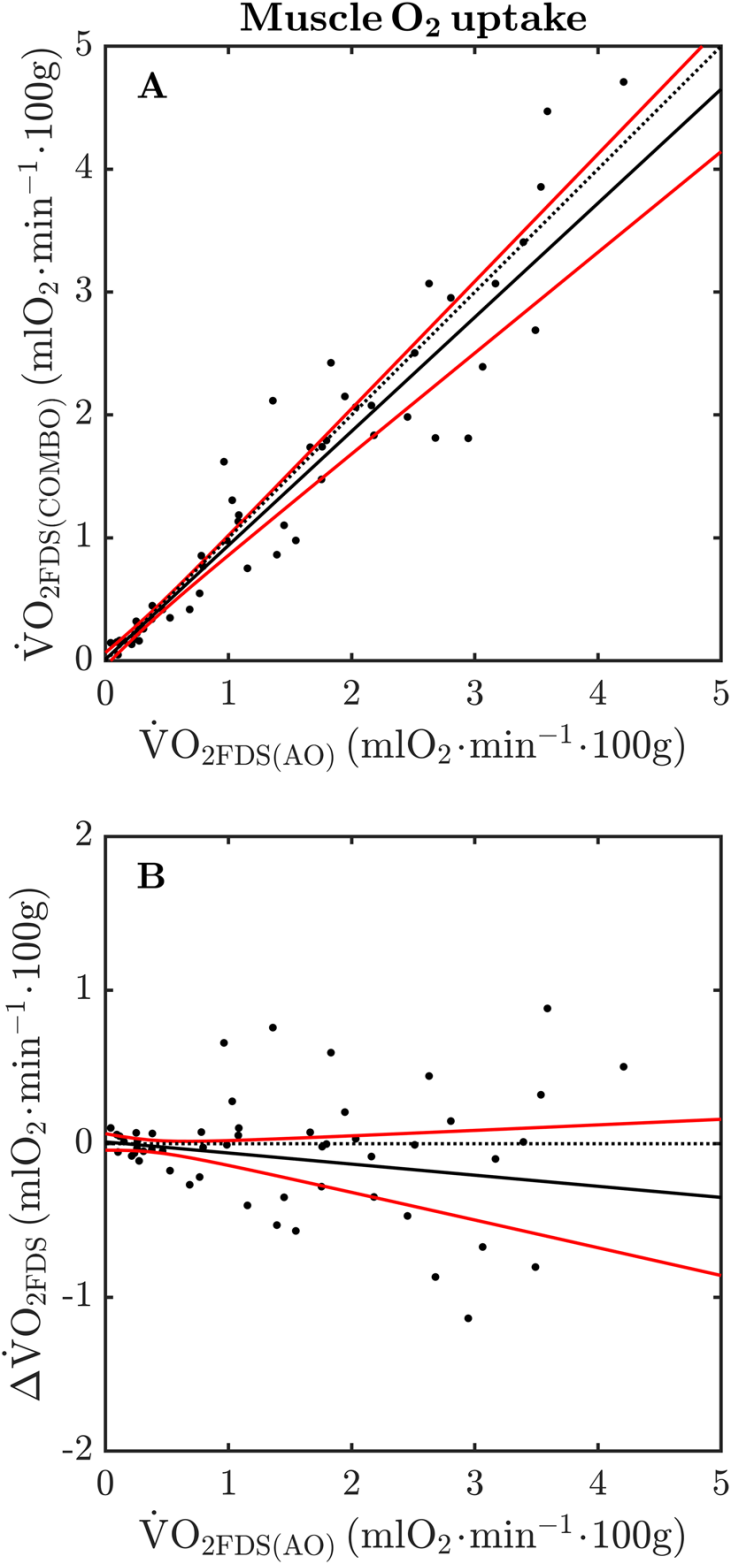
Measurement agreement between arterial and combined occlusion trials for muscle O_2_ uptake (**A**,**B**) of the flexor digitorum muscle (FDS) during isometric handgrip exercise. Solid points represent individual data points from all participants at rest, and immediately following isometric handgrip exercise at 20, 40 and 60% of maximal voluntary contraction. The dotted lines denote the line-of-identity (i.e., perfect agreement). The solid lines represent the predicted group-level response obtained via robust linear mixed effects modeling. The solid red lines represent simultaneous 95% confidence intervals of the predicted group-level response. AO: arterial occlusion; COMBO: combined venous and arterial occlusion; $\Delta  { \dot {\text{V}} \text{O}}_{\text{2}}$: differences in muscle O_2_ uptake AO and COMBO trials, respectively (i.e., COMBO–AO).

The measurement agreement in ${{\dot {\text{V}}}}{{\text{O}}_{2{\text{FDS}}}}$ between AO and COMBO trials across the *entire range* of data is presented in Fig. 5A. It is readily observed from this figure that the absolute agreement in ${{\dot {\text{V}}}}{{\text{O}}_{2{\text{FDS}}}}$ between conditions is much less strong than that observed for ${\dot {\text{Q}}}_{{\text{FDS}}}$ and $\text{C}_{{\text{FDS}}}$ (c.f. Figure 4A,B). For example, the simultaneous CI_95%_ of the robust LME model-fit are relatively wide, and increase in width toward higher values of ${{\dot {\text{V}}}}{{\text{O}}_{2{\text{FDS}}}}$. Despite this observation, it is still worth noting that these confidence intervals encompass the line-of-identity across the entire range of observed values. In a similar manner to that observed for ${\dot {\text{Q}}}_{{\text{FDS}}}$ and $\text{C}_{{\text{FDS}}}$, the slope estimate of the robust LME model was different from zero (*P* < 0.05) whereas the intercept term was not. Based on these findings, we are inclined to state that ${{\dot {\text{V}}}}{{\text{O}}_{2{\text{FDS}}}}$ obtained via the COMBO trial underestimated those values determined from the AO condition by approximately 7%, accompanied by an increasing lack of precision (i.e., wider CI_95%_) at higher values of ${{\dot {\text{V}}}}{{\text{O}}_{2{\text{FDS}}}}$.

## Discussion

The general purpose of this study was to determine whether NIRS-derived measurements of ${\dot {\text{Q}}}_{{\text{FDS}}}$ and ${{\dot {\text{V}}}}{{\text{O}}_{2{\text{FDS}}}}$ could be obtained from a single measurement time point that sequentially combines brief venous followed by arterial occlusions (i.e., the COMBO method). The principal findings of this study were three-fold: (i) ${\dot {\text{Q}}}_{{\text{FDS}}}$, $\text{C}_{{\text{FDS}}}$ and ${{\dot {\text{V}}}}{{\text{O}}_{2{\text{FDS}}}}$ were similar between the relevant occlusion conditions at rest, and immediately following 20, 40, and 60%MVC isometric handgrip contractions; (ii) measurements of ${\dot {\text{Q}}}_{{\text{FDS}}}$ and $\text{C}_{{\text{FDS}}}$ obtained with COMBO trials provided valid estimates of those determined via VO trials across a wide range of physiological values; and (iii) although the measurement agreement for ${{\dot {\text{V}}}}{{\text{O}}_{2{\text{FDS}}}}$ between AO and COMBO trials was fair across the physiological range of values observed in this study, the confidence in agreement (precision) tended to worsen at higher values of ${{\dot {\text{V}}}}{{\text{O}}_{2{\text{FDS}}}}$. Taken together, these findings indicate that our novel COMBO test is a promising approach to assessing both ${\dot {\text{Q}}}_{{\text{FDS}}}$ and ${{\dot {\text{V}}}}{{\text{O}}_{2{\text{FDS}}}}$ simultaneously using NIRS during exercise.

### Muscle blood flow and vascular conductance

Both ${\dot {\text{Q}}}_{{\text{FDS}}}$ and $\text{C}_{{\text{FDS}}}$ systematically increased from rest through to isometric handgrip contractions performed at 60%MVC (Fig. 3). Importantly, however, there were no differences ${\dot {\text{Q}}}_{{\text{FDS}}}$ and $\text{C}_{{\text{FDS}}}$ between VO and COMBO conditions at any level of exercise-intensity. The timing and duration of venous occlusions at rest, and immediately following handgrip exercise were identical between the VO and COMBO trials. Accordingly, it is not surprising that ${\dot {\text{Q}}}_{{\text{FDS}}}$ and $\text{C}_{{\text{FDS}}}$ demonstrated strong absolute (“near-perfect”) agreement between VO and COMBO trials (Table 2 and Fig. 4) given that tissue vasculature has no prescience, and is unable to anticipate the future event of an arterial occlusion (*w.r.t.* COMBO trials). In light of our findings, we are therefore confident that ${\dot {\text{Q}}}_{{\text{FDS}}}$ and $\text{C}_{{\text{FDS}}}$ obtained from COMBO trials are valid surrogates of those measured using the standard VO condition.

### Muscle O_2_ uptake

The ${{\dot {\text{V}}}}{{\text{O}}_{2{\text{FDS}}}}$ response to isometric handgrip exercise was similar between AO and COMBO conditions; namely that ${{\dot {\text{V}}}}{{\text{O}}_{2{\text{FDS}}}}$ systematically increased at higher exercise intensities. That no differences were observed between occlusion conditions at any level of exercise-intensity indicates that, on average, ${{\dot {\text{V}}}}{{\text{O}}_{2{\text{FDS}}}}$ was unaffected by a brief period of venous occlusion. We emphasize that although ${{\dot {\text{V}}}}{{\text{O}}_{2{\text{FDS}}}}$ was similar on the group-level at each exercise-intensity, its level of absolute agreement between AO and COMBO conditions was less impressive than that observed for ${\dot {\text{Q}}}_{{\text{FDS}}}$ and $\text{C}_{{\text{FDS}}}$ (Table 2 and Fig. 4). For example, while CCC values for the relationship between ${\dot {\text{V}} \text{O}}_{{\text{2FDS(AO)}}}$ and ${\dot {\text{V}} \text{O}}_{{\text{2FDS(COMBO)}}}$ indicated near-perfect agreement during exercise at 40 and 60%MVC, these two variables demonstrated only a “substantial agreement” at rest, and during isometric contractions at 20%MVC. As illustrated in Fig. 5A, there was an observable relationship between ${\dot {\text{V}} \text{O}}_{{\text{2FDS(VO)}}}$ and ${\dot {\text{V}} \text{O}}_{{\text{2FDS(COMBO)}}}$ across the entire range of available data. Indeed, the robust LME model indicated a strong linear relationship between ${\dot {\text{V}} \text{O}}_{{\text{2FDS(AO)}}}$ and ${\dot {\text{V}} \text{O}}_{{\text{2FDS(COMB)}}}$ ($R_{{\text{adj}}}^{\text{2}}$ = 97.0). This relationship was best described by a line with negligible offset from the origin (0.01 ± 0.02) and a gradient of 0.93 ± 0.04. In turn, we infer that ${{\dot {\text{V}}}}{{\text{O}}_{2{\text{FDS}}}}$ measured from the COMBO trials tended to *underestimate* those values obtained from the AO (criterion) trials by roughly 7%. Our data do not allow us to comment on potential mechanisms for this underestimation and, as such, this observation remains subject to future investigation. In addition to the above, it is clear that the precision of measurement declines at higher values of ${{\dot {\text{V}}}}{{\text{O}}_{2{\text{FDS}}}}$ (i.e., increasing width of simultaneous CI_9%_ in Fig. 5B). Certainly, the CI_95%_ width of the robust LME model-fit increased from roughly 0.1 mlO_2_ min^−1^ 100 g at the origin to approximately 0.9 mlO_2_ min^−1^ 100 g at the highest observed ${{\dot {\text{V}}}}{{\text{O}}_{2{\text{FDS}}}}$. Based on our findings, we make the following inferences: (i) the relationship between ${\dot {\text{V}} \text{O}}_{{\text{2FDS(AO)}}}$ and ${\dot {\text{V}} \text{O}}_{{\text{2FDS(COMBO)}}}$ displays, at worst, a “substantial” level of absolute agreement; (ii) there is evidence that ${\dot {\text{V}} \text{O}}_{{\text{2FDS(COMBO)}}}$ may slightly underestimate (~ 7%) ${\dot {\text{V}} \text{O}}_{{\text{2FDS(AO)}}}$; and (iii) the measurement precision of ${{\dot {\text{V}}}}{{\text{O}}_{2{\text{FDS}}}}$ determined from the COMBO condition appears to gradually worsen with higher magnitude.

### Practical implications

Our novel combined venous and arterial occlusion (COMBO) protocol offers a significant practical advantage over the VO and AO only protocols; namely that our method provides an estimate of both ${\dot {\text{Q}}}_{{\text{FDS}}}$ and ${{\dot {\text{V}}}}{{\text{O}}_{2{\text{FDS}}}}$ within a *single* measurement period. The time saved by using the COMBO method is exactly half of the time taken to obtain as many measurements of ${\dot {\text{Q}}}_{{\text{FDS}}}$ and ${{\dot {\text{V}}}}{{\text{O}}_{2{\text{FDS}}}}$ via the VO and AO techniques, separately. This time saving affords the investigator with the luxury of choosing to either increase the number of repeated measurements at any given exercise-intensity, or to assess ${\dot {\text{Q}}}_{{\text{FDS}}}$ and ${{\dot {\text{V}}}}{{\text{O}}_{2{\text{FDS}}}}$ over a wider range of work rates than would otherwise be feasible if separate VO and AO trials were performed. Although the COMBO protocol does indeed provide simultaneous estimates of ${\dot {\text{Q}}}_{{\text{FDS}}}$ and ${{\dot {\text{V}}}}{{\text{O}}_{2{\text{FDS}}}}$, it must be conceded that these values are obtained during muscular relaxation and, as such, must differ slightly from those during active contraction. To this end, we acknowledge that recent developments in optical technologies (e.g., diffuse correlation spectroscopy) have enabled the simultaneous and continuous measurement of muscle blood flow and O_2_ uptake during exercise^20–22^. However, these technologies remain prohibitively expensive and are not widely available at present. Until these newer technologies become commonplace, we believe the COMBO method will prove useful to investigators seeking to obtain expedient measurements of ${\dot {\text{Q}}}_{{\text{FDS}}}$ and ${{\dot {\text{V}}}}{{\text{O}}_{2{\text{FDS}}}}$ during exercise.

Although we have demonstrated that the COMBO method provides valid estimates of ${\dot {\text{Q}}}_{{\text{FDS}}}$ and, to a slightly lesser extent, ${{\dot {\text{V}}}}{{\text{O}}_{2{\text{FDS}}}}$ during handgrip exercise, we outline below several improvements to the method that may yet improve its measurement validity.

### Potential improvements

It is known that forearm muscle blood flow progressively declines during a sustained period of venous occlusion^23,24^. We have recently shown that ${\dot {\text{Q}}}_{{\text{FDS}}}$ is highest over the first cardiac cycle following venous occlusion of the forearm^10^. In the present study, however, we were unable to analyze ${\dot {\text{Q}}}_{{\text{FDS}}}$ on a beat-by-beat basis because the update frequency of analog output from the NIRS device was too low (20 Hz). Future implementations of the COMBO method would benefit from measuring NIRS data with a device capable of sampling at higher frequencies (> 50 Hz) to better visualize the arterial pulsatility in the [THb] waveform during the venous occlusion portion of the COMBO method. Following this point, it is conceivable that triggering the onset of venous occlusions to coincide with the R-wave of the electrocardiogram may further improve the reliability of determining ${\dot {\text{Q}}}_{{\text{FDS}}}$ via the COMBO method^25^.

A further caveat to our COMBO method was that venous and arterial occlusions were applied using two separate pneumatic cuffs. Despite our best effort to ensure optimal placement of both cuffs, it was nonetheless likely that the circumferential tension offered by the arterial (outer) cuff was less than optimal. Any “slack” between the forearm and arterial cuff would demand a greater volume of air to charge the cuff-bladder to its target pressure (~ 300 mmHg). A “loose” fitting arterial cuff thus decreases the likelihood that circulatory arrest is achieved within a short period of time (< 0.5 s) which, in turn, distorts the [cHbDiff] waveform in a manner that may ultimately affect the determination of ${{\dot {\text{V}}}}{{\text{O}}_{2{\text{FDS}}}}$. We sought to minimize this slack during COMBO trials by maintaining the inflation of the venous (inner) cuff for an additional 5 s while the arterial (outer) cuff was inflated. It is worth noting that AO trials did not benefit from such an overlapping period of venous cuff inflation. Consequently, the slightly poorer measurement agreement between AO and COMBO trials at high values of ${{\dot {\text{V}}}}{{\text{O}}_{2{\text{FDS}}}}$ (Fig. 5) may be explained, at least in part, by the variable effects of cuff “slack” on the [*c*HbDiff] waveform between the two occlusion protocols. We propose that the problem of cuff “slack” may be circumvented in future studies by interfacing two cuff-inflator systems to a single pneumatic cuff, where rapid “step” changes from supradiastolic (50 mmHg) to suprasystolic pressures (~ 300 mmHg) can be guaranteed.

### Methodological considerations

In the present study, we regarded the values of ${\dot {\text{Q}}}_{{\text{FDS}}}$ and ${{\dot {\text{V}}}}{{\text{O}}_{2{\text{FDS}}}}$ obtained via the VO and AO, respectively, as the “reference” or “criterion” conditions. It must be noted, however, that these measures of muscle blood flow and O_2_ uptake have themselves a degree of measurement uncertainty (i.e., test–retest variability)^9^. It is therefore possible that the inherent variability of our “criterion” measures may have reduced the observed level of measurement agreement between VO and COMBO trials for ${\dot {\text{Q}}}_{{\text{FDS}}}$ (Fig. 4), and between AO and COMBO trials for ${{\dot {\text{V}}}}{{\text{O}}_{2{\text{FDS}}}}$ (Fig. 5). Another consideration of our study is that our numerical approach to determining ${\dot {\text{Q}}}_{{\text{FDS}}}$ and ${{\dot {\text{V}}}}{{\text{O}}_{2{\text{FDS}}}}$ from NIRS waveforms during venous and arterial occlusions (i.e., iteratively reweighted spline fit) does not readily lend itself to the calculation of within-subject variability. We chose to leverage the data obtained from multiple repeat trials to provide a single estimate of the [THb] or [*c*HbDiff] waveform responses to venous and arterial occlusions, respectively. As such, only a single measure of ${\dot {\text{Q}}}_{{\text{FDS}}}$ and ${{\dot {\text{V}}}}{{\text{O}}_{2{\text{FDS}}}}$ is provided from this approach. However, in an effort to provide some measure of test–retest variability within our cohort of participants, we reported the weighted coefficient of variation (CV_w_) of residuals obtained from each spline fit (Table 1). These data indicate that the trial-to-trial variability in [THb] and [*c*HbDiff] waveforms during VO, AO and COMBO trials was less than < 20% during static handgrip exercise. The CV_w_ of these waveforms obtained at rest was much larger, however. These data illustrate that even with 5 repeated measurements obtained at rest (as we have done here), the signal-to-noise ratio is quite poor, and it is likely that a greater number of repeated trials are necessary to reduce the CV_w_ down to a practical range (i.e., < 20%). A final consideration of our study was that we applied the COMBO technique to a relatively small muscle mass (i.e., the FDS muscle) following isometric exercise. What remains to be determined is whether the COMBO method can be applied to larger muscle groups following dynamic exercise.

## Conclusions

This study outlines the development and validation of a novel protocol to measure ${\dot {\text{Q}}}_{{\text{FDS}}}$ and ${{\dot {\text{V}}}}{{\text{O}}_{2{\text{FDS}}}}$ from a single measurement period using NIRS technology. The combined venous and arterial occlusion (COMBO) method provides valid estimates of ${\dot {\text{Q}}}_{{\text{FDS}}}$ and, to a slightly lesser extent, ${{\dot {\text{V}}}}{{\text{O}}_{2{\text{FDS}}}}$ at rest and during static handgrip exercise up to 60%MVC. Notwithstanding the improvements suggested above, the COMBO method provides the investigator with an expedited approach to measuring ${\dot {\text{Q}}}_{{\text{FDS}}}$ and ${{\dot {\text{V}}}}{{\text{O}}_{2{\text{FDS}}}}$ via NIRS during exercise. The time saved by using this approach may then be spent on: (i) obtaining a greater number of repeated trials at a given exercise-intensity; and/or (ii) examining muscle haemodynamics over a wider range of exercise intensities than would otherwise be possible if ${\dot {\text{Q}}}_{{\text{FDS}}}$ and ${{\dot {\text{V}}}}{{\text{O}}_{2{\text{FDS}}}}$ were obtained using separate venous and arterial occlusions.
